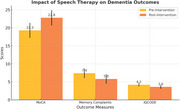# Reconnecting Through Words: Speech Therapy and Dementia

**DOI:** 10.1002/alz70858_102327

**Published:** 2025-12-25

**Authors:** Navilashini Rajasekar

**Affiliations:** ^1^ Freelance speech therapist, Klang, Selangor, Malaysia

## Abstract

**Background:**

The inability to communicate meaningfully hinders dementia patients from connecting with their loved ones. As a key aspect of executive function, communication plays a crucial role in influencing cognitive frailty in dementia patients. Promoting effective and meaningful communication not only fosters a more supportive and enriching environment but also helps preserve cognitive capacity.

**Method:**

Ten individuals diagnosed with mild to moderate dementia participated in a three‐month speech therapy program. The interventions focused on semantic cueing and augmentative communication strategies. Pre‐ and post‐intervention assessments evaluated memory complaints, cognitive function using the MoCA, and caregiver‐reported outcomes through standardized tools, including the IQCODE.

**Result:**

Following the three‐month speech therapy program, participants demonstrated significant improvements in cognitive and functional outcomes. MoCA scores increased significantly from a pre‐intervention mean of 19.3 (SD = 2.1) to 22.8 (SD = 2.5), with a mean difference of +3.5 (t(9) = 4.76, *p* < 0.01). Memory complaints decreased from 7.4 (SD = 1.3) to 5.8 (SD = 1.1), showing a mean difference of ‐1.6 (t(9) = 3.98, *p* = 0.003). Additionally, caregiver‐reported IQCODE scores improved from 4.2 (SD = 0.5) to 3.6 (SD = 0.4), with a mean difference of ‐0.6 (t(9) = 4.22, *p* = 0.002).

**Conclusion:**

The three‐month speech therapy program demonstrated significant improvements in cognitive function, memory complaints, and caregiver‐reported outcomes for individuals with dementia. The marked increase in MoCA scores and the reduction in memory complaints highlight the effectiveness of semantic cueing and augmentative communication strategies in enhancing cognitive and communicative abilities. Additionally, the decrease in caregiver burden, as reflected in IQCODE scores, underscores the broader impact of these interventions on caregiving dynamics. These findings support the potential of structured speech therapy programs to improve the quality of life for both dementia patients and their caregivers, emphasizing their value as an integral part of dementia care.